# Composites in Vehicles Brake Systems-Selected Issues and Areas of Development

**DOI:** 10.3390/ma16062264

**Published:** 2023-03-11

**Authors:** Andrzej Borawski, Grzegorz Mieczkowski, Dariusz Szpica

**Affiliations:** Faculty of Mechanical Engineering, Bialystok University of Technology, 45C Wiejska Str., 15-351 Bialystok, Poland

Modern composite materials, thanks to their excellent properties, are widely used [1,2,3]. One of their applications are friction materials, also used in vehicle brakes [4,5]. Several thousand different components are currently used for the production of composite friction materials, and every day designers consider introducing new ones [6,7]. This is due, among others, to the fact that ecological regulations have been significantly tightened, restricting or prohibiting the use of many of them [8,9]. The best example is asbestos, carcinogenic properties of which have completely eliminated it from the market [10]. It was not an easy task to replace it, which so far has not been a complete success. An ecological challenge is also the fact that the components selected for the production of the composite friction material should be friendly to both the environment and living organisms (terrestrial and aquatic) in three stages: during production, during handling of the finished product, and as a wear product that goes to the surroundings [11,12,13,14].

The ecological aspect is extremely important, but the basic requirement of composites used in braking systems—ensuring the possibility of effective braking of the vehicle [15]—cannot be forgotten. Also, it should be remembered that the brakes work in extremely difficult conditions—fast and large changes in temperature, humidity in the full range of 0–100%, and very high mechanical loads [16,17]. It is also important that the friction materials retain their properties throughout the entire lifetime, which sometimes is even several years [18]. Both the composition itself and the production process have a significant impact on meeting the above-mentioned tasks [19].

As already mentioned, the currently used number of components is very large, and their diversity allows for classification according to various criteria. One of them may be origin, where natural and artificial can be distinguished. Natural materials are necessarily more ecological materials, especially if they are a by-product of another process, such as food production [20,21,22]. They can be of plant origin (stems, leaves), animal origin (fur, hair, shells) or mineral origin (e.g., zeolites) as well as metals and their alloys [23,24,25]. Unfortunately, their mechanical properties and resistance to high temperatures (except for metals) are usually worse than synthetic materials to a greater or lesser extent [26,27,28]. This artificial group of materials includes, above all, various products of the synthesis process, synthetic minerals (e.g., mineral wool), ceramics and others such as carbon and glass fibers [29,30].

Another very important criterion for each component is its function. The following can be distinguished here: matrix, reinforcement, friction modifiers and fillers [31].

The main task of the matrix is to “glue” all the rest. Various types of resins (phenolic, epoxy or silicone) work perfectly in this role [32,33]. The problem may be the fact that the brakes of vehicles, especially those reaching high speeds, heat up to temperatures exceeding the temperature resistance of the resins. Therefore, modifications of their composition are often introduced, which improves their behavior in the above conditions. The matrix, in addition to stability at high temperatures, is required to have good mechanical properties and satisfactory values of the coefficient of friction [34,35]. In the brakes of high-performance vehicles, where the temperature reaches up to 700 °C, this role is taken over by metal matrixes. However, they have their drawbacks—they can cause loud noise or vibrations, and significantly increase the value of the coefficient of friction, mainly due to the tendency to scuffing and adhesion [36,37].

Various types of fibrous materials are excellent as reinforcement. As mentioned, asbestos worked perfectly in this role. Its properties turned out to be difficult to replace [38]. In less loaded systems, this task is easier. For example, plant fibers, shells (of bananas or coconuts), animal hair or cellulose can be used there. The problem increases when more performance is required from the friction composite. Manufacturers then use carbon and glass fibers as reinforcement as well as aramids (including Kevlar). Unfortunately, the problem is that these materials require aggressive chemicals in the production process or can be harmful or irritating themselves [39,40]. The studies in which it was proposed to entrust this role to copper, which must then be in a fibrous form, look promising [41].

One of the most important ingredients are friction modifiers. Their selection and proportions are crucial for an appropriate, equivalent compromise between the value of the coefficient of friction and the coefficient of abrasive wear rate. Hard components, such as steel or cast iron, significantly increase the value of the coefficient of friction. This is because in cooperation with the cast-iron brake disc, adhesion occurs, and as a result, small fragments are “pulled out” [42,43,44]. Unfortunately, this accelerates wear and may cause undesirable braking noises. To reduce these unfavorable properties, the materials are admixed with so-called solid lubricants. They reduce the value of COF and form a thin film on the contact surface, reducing dry friction. Copper works best for this. In addition to lubrication, it perfectly conducts heat, which ensures better heat dissipation from the contact zone [45]. Unfortunately, copper is harmful to both terrestrial and aquatic organisms [46,47]. For this reason, significant limits on its content have been introduced, which are to apply from 2025. Unfortunately, so far no substitute has been found to match the properties of copper. Numerous studies show that graphite is the closest [48].

The last group are fillers. Their role is to fill the empty spaces between the other components [49]. Therefore, materials of this type usually have low price and a fine-grained geometry. Fly ash is the most popular here [50]. As a by-product of combustion, it is a cheap material. Important fact is, that ash is indifferent to the environment. It also does not negatively affect the tribological properties of brake linings, some researchers even show that its high content reduces the maximum temperature achieved during braking.

In recent years, a significant development of composite materials has been noticeable. This gives hope that the composition of the materials for friction linings will be developed, which will meet the more and more restrictive regulations related to ecological aspects, while meeting the increasingly difficult working conditions resulting from the increasing power of internal combustion engines, and the related to it higher accelerations, both at speeding up and braking [51,52,53,54].
